# Physical Fitness Surveillance and Monitoring Systems Inventory for Children and Adolescents: A Scoping Review with a Global Perspective

**DOI:** 10.1007/s40279-024-02038-9

**Published:** 2024-05-06

**Authors:** Javier Brazo-Sayavera, Danilo R. Silva, Justin J. Lang, Grant R. Tomkinson, Cesar Agostinis-Sobrinho, Lars Bo Andersen, Antonio García-Hermoso, Anelise R. Gaya, Gregor Jurak, Eun-Young Lee, Yang Liu, David R. Lubans, Anthony D. Okely, Francisco B. Ortega, Jonatan R. Ruiz, Mark S. Tremblay, Leandro Dos Santos

**Affiliations:** 1 Department of Sports and Computer Science, Universidad Pablo de Olavide, Seville, Spain; 2 Department of Physical Education, Federal University of Sergipe, UFS, São Cristóvão, Brazil; 3 Centre for Surveillance and Applied Research, Public Health Agency of Canada, Ottawa, ON Canada; 4 School of Epidemiology and Public Health, Faculty of Medicine, University of Ottawa, Ottawa, ON Canada; 5 Alliance for Research in Exercise, Nutrition and Activity (ARENA), University of South Australia, Adelaide, SA Australia; 6 Health Research and Innovation Science Centre, Klaipeda University, Klaipeda, Lithuania; 7 Sport Physical Activity and Health Research & Innovation Center (SPRINT), Polytechnic Institute of Guarda, Guarda, Portugal; 8 Faculty of Teacher Education, Art and Sport, Western Norway University of Applied Sciences, Sogndal, Norway; 9 Navarrabiomed, Hospital Universitario de Navarra (HUN), Universidad Pública de Navarra (UPNA), IdiSNA, Pamplona, Spain; 10 Projeto Esporte Brasil, Universidade Federal do Rio Grande do Sul, Porto Alegre, Brazil; 11 Faculty of Sport, University of Ljubljana, Ljubljana, Slovenia; 12 School of Kinesiology & Health Studies, Queen’s University, Kingston, ON Canada; 13 School of Physical Education, Shanghai University of Sport, Shanghai, China; 14 Shanghai Research Center for Physical Fitness and Health of Children and Adolescents, Shanghai, China; 15 Centre for Active Living and Learning, College of Human and Social Futures, University of Newcastle, Callaghan, NSW Australia; 16 Hunter Medical Research Institute, New Lambton Heights, NSW Australia; 17 School of Health and Society, University of Wollongong, Wollongong, New South Wales Australia; 18 Department of Physical Education and Sports, Faculty of Sports Science, Sport and Health, University Research Institute (iMUDS), University of Granada, Granada, Spain; 19 Faculty of Sport and Health Sciences, University of Jyväskylä, Jyväskylä, Finland; 20 CIBER de Fisiopatología de la Obesidad y Nutrición (CIBEROBN), Instituto de Salud Carlos III, Granada, Spain; 21 Instituto de Investigación Biosanitaria, Ibs.Granada, Granada, Spain; 22 Healthy Active Living and Obesity Research Group, CHEO Research Institute, Ottawa, ON Canada; 23 Department of Pediatrics, University of Ottawa, Ottawa, ON Canada

## Abstract

Surveillance of health-related physical fitness can improve decision-making and intervention strategies promoting health for children and adolescents. However, no study has comprehensively analyzed surveillance/monitoring systems for physical fitness globally. This review sought to address this gap by identifying: (1) national-level surveillance/monitoring systems for physical fitness among children and adolescents globally, (2) the main barriers and challenges to implementing surveillance/monitoring systems, and (3) governmental actions related to existing surveillance/monitoring systems. We used a scoping review to search, obtain, group, summarize, and analyze available evidence. Our review involved three stages: (1) identification of surveillance systems through a systematic literature review, with complementary search of the grey literature (e.g., reference lists, Google Scholar, webpages, recommendations), (2) systematic consultation with relevant experts using a Delphi method to confirm/add systems and to gather and analyze information on the barriers and challenges to implementing systems, and (3) Web searches for public documents on government sites and surveillance/monitoring system pages, and direct internet searches to identify relevant governmental actions related to surveillance systems. A total of 15 fitness surveillance/monitoring systems met our inclusion criteria. Experts identified a lack of government support and funding, and the low priority of fitness on the public health agenda as the main barriers/challenges to implementation. Several governmental actions related to surveillance systems were identified, including policies, strategies, programs, and guidelines. We propose a Global Observatory of Physical Fitness to help address these issues.

**Supplementary Information:**

The online version contains supplementary material available at 10.1007/s40279-024-02038-9.

## Key Points

**Table:** 

Few countries have implemented a national surveillance/monitoring system for physical fitness among children and adolescents.
The main barriers/challenges to implementing a surveillance/monitoring system for physical fitness among children and adolescents are related to government support, funding, and the low priority of fitness on the public health agenda.
Countries with surveillance/monitoring systems have developed policies, strategies, and programs or have created guidelines linked to their surveillance/monitoring systems.

## Introduction

Physical fitness is a set of physical attributes that helps people perform physical activities and everyday tasks [1]. Physical fitness is often categorized into skill-related (i.e., agility, balance, speed, coordination, power, and reaction time) and health-related (i.e., cardiorespiratory fitness [CRF], musculoskeletal fitness [MSF], and body composition) components [2, 3], the latter of which are considered health-related because they are important markers of current and future health [4, 5]. The health benefits of these markers are well documented for children and adolescents and provide the basis for fitness surveillance to inform decision-making [6]. For example, higher levels of CRF are associated with favorable health outcomes, including lower adiposity, improved cardiometabolic health, cognitive function, and mental health, among children and adolescents [7–10]. Higher levels of MSF are associated with a lower adiposity, reduced cardiovascular and metabolic disease risk, and better academic outcomes [11–14]. Furthermore, CRF and MSF are associated with a better quality of life among children and adolescents [15].

Childhood and adolescence are periods of physical and behavioral change that can directly impact future health [16, 17]. Encouraging active lifestyles, such as increased physical activity and sport participation, while preventing extended time spent being sedentary, is part of the global agenda for adolescent health [18]. However, progress in promoting physical fitness as a marker of health has been uneven and inconsistent [19]. A recent Delphi study of international experts identified the top 10 research and surveillance priorities for physical fitness among children and adolescents. The top three priorities were the development of longitudinal studies that measure changes in physical fitness and their relationship with health, the use of surveillance systems to inform decision-making, and the implementation of regular and consistent international/national fitness surveys using common measures [6]. These priorities are related, as data from surveillance systems can provide more accurate estimates of longitudinal changes in physical fitness through regular assessments, which can facilitate decision-making and improve mid- and long-term intervention strategies [6].

To understand surveillance systems, in this review, we make the following distinctions: *fitness surveillance*, which is linked to a governmental agency or public policy, differs from *fitness monitoring*, which regularly evaluates fitness levels over time but does not necessarily influence public policy, and *fitness testing*, which involves cross-sectional or longitudinal assessments of physical fitness within the same individuals [20]. In this sense, Japan is an example of how physical fitness surveillance can impact decision-making regarding physical activity policies, because it is a government initiative with a long tradition and it has been improved since 1964 [21]. Surveillance systems are crucial to public health as they provide data for developing health services and policies, including identifying changes in physical fitness levels associated with risk factors and healthy practices [20].

There are ongoing projects that target informing about youth physical fitness such as the Active Healthy Kids Global Alliance Global Matrix initiative (https://www.activehealthykids.org), which identifies and groups current information on physical activity-related indicators. In its last edition, physical fitness could not be graded in 54% of countries/territories (31/57) because data were poor or lacking [22]. The low number of countries with fitness data highlights the need for feasible and scalable measures to better facilitate the global surveillance of physical fitness. Additionally, there are other initiatives for monitoring fitness, such as the European Network for Supporting the Development of Physical Fitness Monitoring Systems for Children and Adolescents (FitBack) and the European Fitness Monitoring System (EUFITMOS); these are European-wide initiatives that aim to encourage physical activity participation and to create a network for monitoring youth fitness levels [23, 24]. Despite these initiatives, no global surveillance system exists that provides a comprehensive understanding of physical fitness levels and trends. Furthermore, while the physical fitness monitoring systems in Europe were reviewed as part of the FitBack proposal [25], the number and geolocation of national surveillance systems worldwide is unknown.

Therefore, the research questions of this review are as follows: What national surveillance systems for physical fitness of children and adolescents exist and what are their main characteristics? What are the main challenges and barriers to the development and implementation of these systems? Are these systems used to inform public health systems and policies? Answers to these questions may enhance our understanding of the lack of global data on the systematic assessment of physical fitness among children and adolescents and the differences among surveillance procedures, such as the types of fitness tests used, their comprehensiveness, and the frequency of assessments. Identifying the main challenges to and facilitators of implementation can help map strategies to improve existing systems and promote surveillance in countries and regions where they are less frequent or non-existent. The aims of this scoping review were to (1) identify national-level surveillance/monitoring systems for physical fitness among children and adolescents globally, (2) identify the main barriers and challenges to implementing surveillance/monitoring systems, and (3) identify governmental actions associated with existing surveillance/monitoring systems.

## Methods

### Study Design

We conducted a scoping review to search, obtain, group, summarize, and analyze available evidence using the Preferred Reporting Items for Systematic Reviews and Meta-Analyses Extension for Scoping Reviews (PRISMA-ScR) checklist [26]. The review was divided into three stages: (1) systematic literature review, with complementary searches of the grey literature (e.g., study reference lists, Google Scholar, webpages, recommendations) to identify surveillance systems; (2) systematic consultation with relevant experts using a Delphi method to confirm/add systems and to gather and analyze information about the barriers and challenges to implementing systems; and (3) Web searches for public documents on government and surveillance/monitoring system pages, and direct internet searches to identify associated governmental actions related to surveillance systems.

### Identifying Surveillance/Monitoring Systems

#### Information Sources

To identify national surveillance/monitoring systems for physical fitness among children and adolescents and experts in this field in different parts of the world, we developed a two-step search strategy: (1) a systematic search for studies that used evidence from surveillance/monitoring systems with measures of physical fitness and a review of the corresponding reference lists, and (2) a keyword search (surveillance system, physical fitness, child, adolescents) of webpages for systems.

#### Search

The searches were performed in Medline (via PubMed). The complete search strategy is available in the Electronic Supplementary Material Appendix S1, along with the protocol in the Open Science Framework (OSF) platform [27]. Keywords related to systems, physical fitness, and children and adolescents were used, combining Medical Subject Headings (MeSH) terms and common descriptors. Terms were combined using the Boolean operators *AND*, *OR*, and *NOT*. We searched PROSPERO, the Cochrane Library, and the reference lists of included articles.

We also directly searched the grey literature through Google Scholar, using simple keywords such as *surveillance system*, *physical fitness*, and *children/adolescents*, with the first 500 results screened.

#### Selection of Sources of Evidence

The following inclusion and exclusion criteria were used for screening:

*Inclusion*:

- Surveillance or monitoring systems for school-aged children and adolescents aged between 5 and 17 years
- Surveillance or monitoring systems using a nationally representative sample
- Surveillance or monitoring systems that included at least measures of CRF and MSF

*Exclusion*:

- Physical fitness surveillance or monitoring systems specific for children or adolescents with pathologies (e.g., asthma, cancer, other conditions)
- Surveillance or monitoring systems at the international or regional level, and/or providing only local data
- Surveillance or monitoring systems based only on questionnaires or interviews
- Cross-sectional, cohort, or interventional studies

#### Study Screening and Selection

Electronic database search results were exported into Rayyan [28], where duplicates were removed. Two independent researchers (LDS and DRS) screened titles and abstracts for eligibility. The results were compared, and conflicts were resolved in consultation with a third researcher (JB-S). The full texts of eligible studies were then reviewed for eligibility. The authors were contacted if more information was needed to determine eligibility. The same two independent researchers (LDS and DRS) screened full texts for eligibility, with conflicts resolved by a third researcher (JB-S).

#### Data Charting and Extraction

The data extraction sheet was piloted using the five surveillance/monitoring systems to ensure consistency, with modifications made and documented. Data were extracted from the following domains: information about the system, information about the sample, measurements, and tests used. Information about the surveillance/monitoring system included: name and acronym (if any), country, start/end years, sampling process, age range, physical fitness tests used, and frequency of assessments.

### Consultation with the Panel of Experts

Experts and researchers with experience in surveillance/monitoring systems for physical fitness among children and adolescents were consulted to confirm and/or add to the systems identified in the literature (see Electronic Supplementary Material Appendix S2 for the list of international experts). They were asked to identify the main barriers and challenges to implementing physical fitness surveillance/monitoring systems. For both purposes, the Delphi method (a structured communication technique that aims to gather opinions on a particular research question or specific topic to gain consensus) was used [29].

#### Identification of Experts

We selected a stratified panel of experts from the initial search using the World Health Organization (WHO) regional classification (i.e., Africa, Americas, South-East Asia, Europe, Eastern Mediterranean, and Western Pacific). We identified at least two experts from each region. For eligibility, the expert had to meet at least one of the following criteria: (1) coordinated or collaborated in the development of a surveillance/monitoring system for physical fitness among children and adolescents, or (2) published paper(s) using data from surveillance/monitoring systems for physical fitness among children and adolescents in their region.

#### Survey Development

A survey comprising closed and open questions was developed to gather information from experts on surveillance/monitoring systems that had not been captured by the literature search. The complete set of questions can be accessed in Electronic Supplementary Material Appendix S3.

#### Delphi Round 1: Survey Distribution

In the online questionnaire, experts were asked to indicate known surveillance/monitoring systems and the barriers and challenges to implementation.

#### Round 2: Feedback from Experts

Experts were provided with a summary of round 1 findings, where ten barriers and challenges were identified. Using a Likert scale, ranging from 1 (totally disagree) to 5 (totally agree), the experts indicated the degree of importance of each barrier and challenge. The results of round 2 were used to determine consensus among the experts’ responses and rank barriers and challenges according to the priority indicated in the responses. We used Malhotra and colleagues’ (2011) [30] calculation for mean ranking (Eq. 1):

1 $$Mean\,ranking= \frac{Strongly\,Agree + 5 \times Agree + 4 \times Neutral + 3 \times Disagree + 2 \times Strongly\, Disagree + 1}{Strongly\,Agree + Agree + Neutral + Disagree + Strongly\,Disagree}$$

### Governmental Actions Associated with Surveillance Systems

To identify governmental actions associated with surveillance systems, additional searches were performed in the grey literature, the webpages of the systems themselves, and via email consultation with technical and/or scientific managers. Our grey literature search was conducted in English, Portuguese, and Spanish, but no language restrictions were set for material sent from consultations. The system managers were identified using the contact information provided on the corresponding websites, and additionally, experts collaborating with the current study from some countries involved were consulted when the action was in a different language. This information was considered official. This step achieved the third aim and provided the link between the identified surveillance systems and the actions developed.

## Results

### Surveillance/Monitoring Systems

**Fig. 1:**
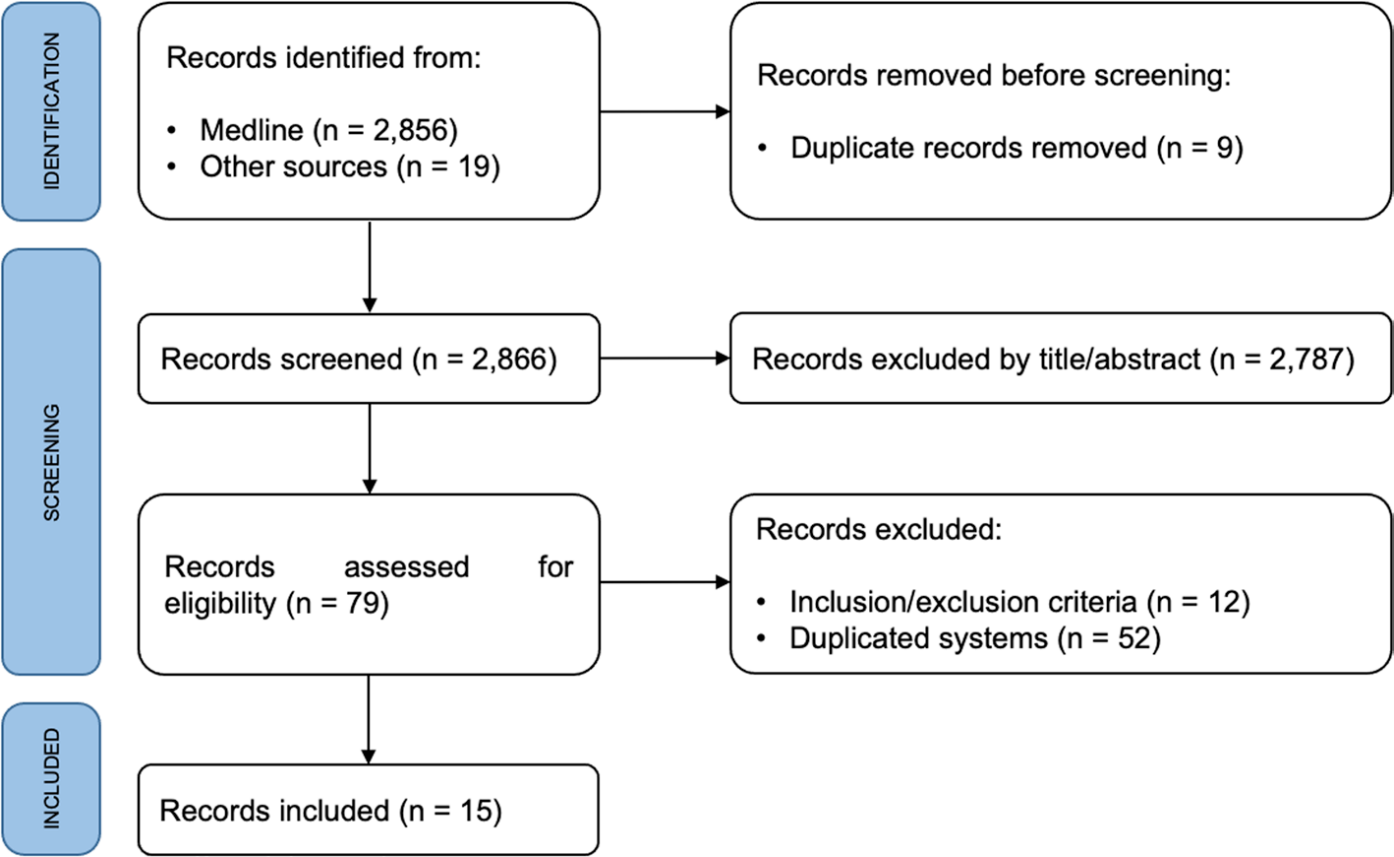
Surveillance systems search flow diagram

Figure 1 depicts the flowchart of surveillance/monitoring system searches. The systematic literature review resulted in 2856 studies, with 19 additional studies located through the grey literature and expert suggestions. After removing duplicates, 2866 titles and abstracts were screened for eligibility. A total of 79 studies were eligible for full-text screening. Among them, 52 studies (some surveillance/monitoring systems, data sets, or fitness repositories) were duplicates and 12 systems did not meet the inclusion criteria. Finally, 15 items met the eligibility criteria. Electronic Supplementary Material Appendix S4 describes the excluded systems and the reasons for exclusion.

Table 1 describes the 15 included national surveillance/monitoring systems. Nearly half of the surveillance/monitoring systems were from Europe (*n* = 7), followed by Asia (*n* = 4), North America (*n* = 2), and South America (*n* = 2). The oldest system began in China in 1954, and the most recent was the Lithuanian system, which planned to start in 2019 and was delayed due to the coronavirus disease 2019 (COVID-19) pandemic. The age range of participants was 3 years and above. A total of 74 different tests were used to assess physical fitness, including 13 for CRF, 23 for MSF, seven for speed, 11 for agility, six for flexibility, 13 for body composition, and one for balance. All systems performed one annual measurement, except for the Serbian system, with two measurements per year, and the Canadian Health Measures Survey (CHMS), with samples accumulated every 2 years.

**Table 1:** Surveillance and monitoring systems included

Title	Acronym	Country	Start year	Final year	Sampling process	Age range	Measures of physical fitness	Frequency
National Standard of Physical Fitness of Students	NSPFS	China	1954	Present	Compulsory for all students (primary school [grade 1–6], junior high school [grade 7–9], high school [grade 10–12]) and college/university undergraduate students) in China	6–22	8 × 50-m shuttle run, 800- (girl)/1000-m (boys) endurance running, forced vital capacity of lung (spirometry), standing long jump, oblique body pull-ups, pull-ups (boys), 60-s sit-ups (girls), 50-m dash, 60-s rope-skipping, sit-and-reach, height, weight, BMI	Annual
Japan Fit Survey for All Ages	-	Japan	1964	Present	School-aged children (aged 6–17 years) randomly sampled from each of Japan’s 47 prefectures, with 50–60 students from each age–gender group selected from participating schools	6–79	20-m shuttle run, handgrip strength, standing long jump, sit-ups, softball throw or handball throw, 50-m sprint, sit-and-reach	Annual
National surveillance system for physical and motor development of children and youth in Slovenia	SLOfit	Slovenia	1982	Present	SLOfit has enabled annual monitoring of 220,000 students aged 6–19, making it possible to measure almost the entire Slovenian population every April	6–19	600-m run, standing long jump, 60-s sit-ups, bent arm hang, 20-s arm plate tapping, polygon backward, 60-m dash, stand-and-reach, height, weight, triceps skinfold	Annual
Brazil Sport Project	PROESP-Br	Brazil	1994	Present	The system includes data from schoolchildren from schools that voluntarily adopt the battery of tests that make up PROESP and form a national database of physical fitness monitoring	6–17	6-min run/walk, standing long jump, curl-ups, medicine ball chest pass, 4 × 4-m agility test, 20-m run, sit-and-reach, height, weight, BMI, waist circumference, wingspan	Annual
National Health and Nutrition Examination Survey	NHANES	USA	1999–2000	Present	NHANES used four stages of sampling to select participants	3–oldest	Cardiorespiratory fitness (treadmill), handgrip strength, height, weight, waist circumference, DEXA skinfolds	Annual
Canadian Health Measures Survey	CHMS	Canada	2007–2009	Present	CHMS uses a stratified 3-stage sampling method to select one or two respondents from each dwelling in a sampled collection site, with strata defined at each stage based on geographic regions, household composition, and age groups	3–79	Cardiorespiratory fitness (mCAFT), handgrip strength, jumping mechanography testing using the Leonardo mechanograph ground reaction force plate, sit and reach, height, weight, BMI, waist circumference, skinfolds	Biannual
National System of Evaluation of Learning Outcomes of the Ministry of Education of Chile on Physical Education	SIMCEEF	Chile	2010	2011	Population-based system that assesses all 8th grade students in Chile	13–14	20-m shuttle run, Cafra test, standing long jump, sit-ups, push-ups, sit-and-reach, height, weight, BMI, waist circumference	Annual
French national program to assess the physical fitness, organized by *Institut des Rencontres de la Forme*	DIAGNOFORM	France	2010	2018	Data from several regions of France. All schools in France were invited to participate in the study, with each school director deciding whether to participate	5–11	20-m shuttle run, standing long jump, running as fast as possible for 5 s, test measuring the capability to reach down as far as possible starting from standing position and maintaining the position for 3 s (flexibility)	Annual
Physical Activity Promotion System	PAPS	South Korea	2010	Present	PAPS is conducted in all public and private schools in South Korea to evaluate physical fitness and provide exercise-related guidance to elementary, middle, and high school students	9–18	15-m and 20-m shuttle run, standing long jump, handgrip strength, muscular strength, sit-and-reach test, height, weight, BMI	Annual
National Fitness Award	NFA and NFA for early years	South Korea	2011	Present	This is a government-operated program that offers free fitness testing services to individuals who willingly participate in 75 testing centers located throughout the country. Initially designed for individuals aged 11 years and older, a pilot program for children aged 4 and 6 years commenced in 2023. The pilot program was based on a stratified sampling method by age, sex, and area of residence	4–6 years; 11 years–oldest	*4–6 years:* standing long jump, handgrip strength, curl-up, 10-m shuttle run, 4 × 5-m shuttle run, 3 × 3 button-pushing, sit-and-reach, height, weight *11–12 years:* 15-m shuttle run, standing long jump, handgrip strength, partial curl-ups, side run, coordination 3 × 3 button push, eye-hand coordination, sit-and-reach, height, weight, BMI, weight-height ratio, body fat measures (bioelectric impedance) *13–18 years:* 20-m shuttle run, treadmill/step testing for $\dot{V}$ O_2max_, standing long jump, handgrip strength, partial curl-ups, repeated jump test, flight time, Illinois agility test, eye-hand coordination, sit-and-reach, height, weight, BMI, body fat measures (bioelectric impedance)	Annual
Hungarian National Student Fitness Test	NETFIT	Hungary	2013	Present	NETFIT used a nationally representative sample of 2686 participants, selected from 53 schools across seven regions using 2-stage stratified random sampling. A subsample of 578 was randomly selected for lab tests in five regional laboratories	11–19	Handgrip strength, standing long jump, push-ups, curl-ups, trunk lifts, modified sit-and-reach, body fat measures (bioelectric impedance)	Annual
A national physical functional capacity monitoring and feedback system for Finnish students in grades 5 and 8	Move!	Finland	2016	Present	Population-based system composed of students in grades 5 and 8	11–14	5 continuous jumps, upper body lift, push-ups, squats, throw-catch combination (handling skills, perceptual motor skills, upper limb strength), lower back extensions, 20-m line run, shoulder mobility	Annual
FitEscola	FitEscola	Portugal	2016	Present	The system includes all Portuguese schoolchildren		20-m shuttle run, 1-mile walk, long jump, horizontal jump, sit-ups, push-ups, 10-m agility shuttle run, shoulder mobility test, sit-and-reach, BMI, triceps and calf skinfolds, bioelectrical impedance, waist circumference	Annual
National fitness monitoring system in Serbia	-	Serbia	2017	Present	Population-based system which tests all students in the age range	9–19	20-m shuttle run, standing long jump, bent arm hang, 30-s sit-ups, 4 × 10-m shuttle run, sit-and-reach, height, weight, BMI	Semestral
Lithuanian physical fitness monitoring system	-	Lithuania	2019	Present	The system includes all students from grades 1 to 12 in all general education schools	6–17	20-m shuttle run, 6-min run, standing long jump, tennis ball throw, bent arm hang, 10 × 5-m shuttle run, flamingo (balance), sit-and-reach	Annual

CHMS: The Canadian system uses different tests every cycle. NFA: Evaluates sporadically across the year. The Lithuanian physical fitness monitoring system has not yet started due to the COVID-19 pandemic

*BMI* body mass index, *COVID-19* coronavirus disease 2019, *DEXA* dual-energy X-ray absorptiometry, *mCAFT* modified Canadian Aerobic Fitness Test, $\dot{V}$ *O*_*2max*_ maximal oxygen consumption

#### Challenges to Implementing Physical Fitness Surveillance/Monitoring Systems

Table 2 shows the main barriers/challenges to the development of surveillance systems for physical fitness among children and adolescents indicated by the experts, ranked according to their responses in round 2. Among the ten barriers/challenges listed in round 1, the top three (most challenging) were (1) lack of government support, (2) lack of funding, and (3) low priority of fitness on the public health agenda. The bottom three (least challenging) were difficulties with human resources, ethical and legal problems, and the gaps in the relevant literature.

**Table 2:** Ranking of challenges and barriers to the development of physical fitness surveillance/monitoring systems for children and adolescents according to the responses of the expert panel (*n* = 16)

Main barriers and challenges	Rank	Mean ranking
Government support (convincing governments and decision makers to employ national surveillance systems in the country)	1	3.2
Financial support (lack of financial resources, funding, grants)	2	3.1
Public health priority (it is not considered a public health priority agenda)	3	2.9
Logistical support (data collection and managing data entry from multiple sources)	4	2.7
Schools support (barriers to obtaining collaboration from managers and schools)	5	2.6
Participation rate (having difficulty achieving adherence in some countries)	6	2.2
Standardization of tests (need to standardize tests to allow comparison)	7	1.8
Human resources (difficulty in training the work team and physical education teachers)	8	1.7
Ethical and legal (personal data protection legislation and political and cultural barriers)	9	1.5
Lack of literature on physical fitness (lack of substantial results on physical fitness related to the health of children and adolescents)	10	1.2

#### Governmental Actions Associated with Surveillance Systems

Table 3 presents the governmental actions related to the identified surveillance systems. These policies were developed at the national or local level and were informed by surveillance data. Some examples of government actions supported by surveillance systems include the development of Physical Activity Guidelines, National Strategies for the Promotion of Physical Activity, and internal Ministry reports on physical fitness. These actions may be designed to encourage physical activity, provide recommendations and guidelines for promoting physical fitness, and/or inform government decision-making. The specific goals of these actions may have varied, but they all aimed to promote the physical health and well-being of children and adolescents.

**Table 3:** Governmental actions linked to the surveillance systems identified

Surveillance system	Acronym	Country	Governmental actions and/or related system information
National Standard of Physical Fitness of Students	NSPFS	China	Internal report is written based on findings from NSPFS and submitted to the Ministry of Education of China annually. The Ministry of Education summarized some key messages and provided them to the State Council of China. Public policies were subsequently released in response. This system includes the “Physical Activity and Fitness in China—The Youth Study” (PAFCTYS) that started in 2016
Japan Fit Survey for All Ages	-	Japan	Conducted by the Japan Sports Agency (Ministry of Education, Culture, Sports, Science, and Technology until 2015) to provide data for public policies
National surveillance system for physical and motor development of children and youth in Slovenia	SLOfit	Slovenia	Slovenian educational policy, informed by the SLOfit data, managed to develop the systems of physical education and extracurricular sports programs: https://www.gov.si/en/policies/education-science-and-sport/ (accessed 06/09/2023) National strategy for nutrition and physical activity 2015–2025. Available at http://pisrs.si/Pis.web/pregledPredpisa?id=RESO101# (accessed 06/09/2023) National program of sport 2014–2023. Available at http://www.pisrs.si/Pis.web/pregledPredpisa?id=RESO93# and https://e-uprava.gov.si/drzava-in-druzba/e-demokracija/predlogi-predpisov/predlog-predpisa.html?id=4362 (accessed 06/09/2023)
Sport Brazil Project	PROESP-Br	Brazil	Linked to government agencies: Second time program (Programa Segundo Tempo). Available at https://www.ufrgs.br/proesp/historico.php (accessed 06/09/2023)
National Health and Nutrition Examination Survey	NHANES	USA	The NHANES can inform the development and implementation of policies aimed at promoting physical activity and improving the health and fitness of children and adolescents. For example, the Physical Activity Guidelines for Americans. Available at https://www.cdc.gov/nchs/nnyfs/index.htm and https://www.cdc.gov/nchs/data/series/sr_02/sr02_163.pdf (accessed 06/09/2023)
Canadian Health Measures Survey	CHMS	Canada	CHMS data support the development of recommendations and guidelines to promote physical fitness and physical activity in children and adolescents in Canada. Participaction: https://www.participaction.com/the-science/children-and-youth-report-card/ (accessed 06/09/2023). Canadian Physical Activity Guidelines: https://cdnsciencepub.com/doi/pdf/10.1139/H11-009 (accessed 06/09/2023)
National System of Evaluation of Learning Outcomes of the Ministry of Education of Chile on Physical Education	SIMCEEF	Chile	The SIMCEEF national surveillance system was designed to improve the quality of physical education classes and assess the physical fitness of students in the eighth cycle as part of Chile’s national education quality control policy. The system is associated with the Chilean Education Quality Agency. By utilizing SIMCEEF, it can strive to enhance the overall quality of physical education classes and promote better physical fitness among students. Available at https://www.agenciaeducacion.cl/informar/estudios/estudios-nacionales/ (accessed 06/09/2023)
French national program to assess the physical fitness, organized by IRFO	DIAGNOFORM	France	As part of the IRFO, DIAGNOFORM provides information on the physical fitness of the French population, which is used for the development of actions to promote physical activity. Available at https://irfo.fr/decouvrez-lirfo/ (accessed 06/09/2023)
National Fitness Award	NFA and NFA for early years	South Korea	It is operated by the Korea Sports Promotion Foundation under the *Citizen’s Health Promotion Act* (Sect. 16.2), a government welfare program aimed at accomplishing two main goals: (1) encouraging higher engagement in physical activity and (2) providing tailored physical activity programs based on scientific evaluation. Additionally, FA serves as one of the employment criteria for physically demanding occupations. Today, NFA has its own social media channels for knowledge translation efforts (e.g., Instagram: https://www.instagram.com/national_fitness_award/; YouTube: https://www.youtube.com/channel/UCpjBiFyCh3f5bDU99Izt8Fw). Available at https://nfa.kspo.or.kr/main.kspo (accessed 06/09/2023)
Physical Activity Promotion System	PAPS	South Korea	Not reported
Hungarian National Student Fitness Test	NETFIT	Hungary	Annually collected data serves as a foundation for planning physical education classes and guiding actions at a national level. Available at https://www.netfit.eu/public/pb_about.php (accessed 06/09/2023) and https://net.jogtar.hu/jogszabaly?docid=a1200020.emm (accessed 06/09/2023)
A national physical functional capacity monitoring and feedback system for Finnish students in grades 5 and 8	Move!	Finland	The Move! system was developed in cooperation with the Ministry of Social Affairs and Health, the National Institute for Health and Welfare, and the Trade Union of Education in Finland. On the national level, Move! produces objective information about children’s and adolescents’ physical functional capacity, for example, to support political decision-making. Available at https://www.oph.fi/en/education-and-qualifications/move-monitoring-system-physical-functional-capacity and https://www.oph.fi/en/education-and-qualifications/move-what-it-why-it (accessed 06/09/2023)
FitEscola	FitEscola	Portugal	FitEscola contributes to the National Strategy for the Promotion of Physical Activity, Health, and Well-Being, which provides for the preparation of an intersectoral action plan, which includes the possibility of monitoring the levels of physical fitness of children and young people. Available at https://www.cns.min-saude.pt/wp-content/uploads/2018/12/GERACOES-MAIS-SAUDAVEIS.pdf (accessed 06/09/2023)
Serbian surveillance system	-	Serbia	Not reported
Lithuanian physical fitness monitoring system	-	Lithuania	It is part of the public policy of the Ministry of Health of the Republic of Lithuania. Available at https://e-seimas.lrs.lt/portal/legalAct/lt/TAD/d43a6300ebf211e99ab7ff5a9ea34fcc (accessed 06/09/2023)

*FA* Fitness Award, *IRFO* Institut des Rencontres de la Forme

## Discussion

To our knowledge, this review is the first to provide a comprehensive overview of surveillance/monitoring systems for physical fitness among children and adolescents globally. We identified 15 national physical fitness surveillance/monitoring systems for children and adolescents worldwide, located in upper-middle- and high-income countries in the Americas, Asia, and Europe. The main barriers/challenges identified for the implementation of national surveillance/monitoring systems among children and adolescents were lack of government support and funding and the low priority of fitness on the public health agenda. Additionally, most of the systems identified were associated with governmental actions.

These results highlight the limited number of national surveillance/monitoring systems available for physical fitness among children and adolescents, particularly in low- and middle-income countries. Australia and Africa were the only continents without a national surveillance system. Similar data gaps in low- and middle-income countries were also observed with physical activity surveillance for children and adolescents [31, 32]. In the case of Australia, the first national survey of health and fitness for children and adolescents was conducted in 1985. However, this survey was never repeated. Since then, repeated cross-sectional surveys up until 2015 have collected representative state-based fitness data on Australian children and adolescents [33]. In Africa, the lack of physical fitness surveillance systems can be attributed to additional factors, which tend to be shared among low-income countries. First, there is a lack of resources, including trained personnel, equipment, and funding, which limits the implementation and sustainability of such systems. Second, cultural, and social norms may not prioritize physical activity, leading to a lack of interest and participation in fitness monitoring programs, with the prevention of infectious diseases one possible priority displacing such efforts. Third, the diversity of contexts and the communication between them require tailored approaches to physical fitness monitoring, which can be difficult to develop and implement at scale. The lack of comprehensive surveillance/monitoring systems presents a challenge to understanding the current levels of physical fitness among youth and implementing targeted interventions. This challenge has significant implications for global physical fitness, as low- and middle-income countries represent a large proportion of the world’s population. Addressing physical fitness disparities is important for achieving global health equity, given the positive association between improved physical fitness and overall health outcomes. To address gaps in surveillance and monitoring systems, it is essential to establish robust routine health information. Also, further research is required to develop solutions and potentially support and facilitate fitness surveillance capacity building efforts on a global scale [34].

The diversity of approaches and procedures observed in the selected surveillance/monitoring systems is noteworthy, considering the limited number of available systems. The use of 74 different tests highlights the challenge of comprehensively measuring and evaluating physical fitness. Among these tests, body composition, CRF, and MSF were the most tested components, supporting their legitimacy as markers of physical and mental health. The most common tests included the 20-m shuttle run and 6-min walk/run for CRF, and handgrip muscle strength, standing long jump, push-ups, and bent arm hang for MSF. Balance, flexibility, speed, agility, and coordination were also measured, albeit with fewer tests. With a variety of tests available, it can be challenging to establish a standard protocol for universal fitness surveillance [35]. The absence of standardized protocols and the wide range of tests employed across different systems may hinder direct comparisons and the establishment of global benchmarks for physical fitness in children and adolescents. Moving forward, it will be important to develop consensus guidelines, as well as globally valid and reliable approaches to ensure the consistency and comparability of physical fitness surveillance data worldwide. Standardization would not only facilitate meaningful comparisons between countries but also enable the identification of trends and the development of effective interventions to improve the physical fitness levels of children and adolescents globally. There are several physical fitness test batteries that could be used to provide a standard method of assessment [35].

The top three barriers/challenges identified by the experts are related to political priorities, including government support, financial support, and public health priority. The increasing body of literature on physical fitness surveillance also suggested a gap between research and practice [6]. Lessons learned from other projects provide promising solutions to reduce this gap, such as creating awareness of the importance of fitness surveillance among youth, reducing bureaucratic barriers to implementation of the systems, or providing stable funding [36]. The FitBack initiative proposes solutions to establish physical fitness surveillance systems, including the implementation of national school-based physical fitness surveillance/monitoring systems for children and adolescents [25]. Although this European proposal tries to facilitate the work for different countries in the world, it is important to highlight that different approaches perhaps should be considered for countries in other world regions as there are different economic, social, and political situations to be addressed.

Most systems identified were conducted annually and were associated with governmental actions promoting physical activity and well-being. Including physical fitness surveillance as a state/government policy remains crucial for long-term sustainability and integrating fitness data into decision-making processes. Furthermore, in most countries, the responsibility for conducting annual physical fitness surveillance is legislated. Legal determinants of health [37] refer to the fundamental aspects outlined in a country’s legal framework that have an impact on the health and well-being of its population. These determinants vary across countries, but generally include the right to health, social welfare, non-discrimination, access to clean water, and sanitation. As these determinants provide a legal framework and guiding principles to shape policies, programs, and actions aimed at improving public health and well-being, it is important to encourage countries to follow the legislative process for their physical fitness surveillance and monitoring efforts. Besides surveilling physical fitness at the population level, there is an opportunity to increase its clinical use, given the links between youth fitness levels and current and future health [38]. However, further investigation is needed to better understand how the systems are used by policymakers and how the systems can be improved to communicate with intersectoral policies, such as in health, education, and sport.

The Global Observatory of Physical Activity was launched to address the lack of standardization in physical activity surveillance and reduce the insufficient physical activity practice across the globe [39]. Since then, other initiatives have been implemented worldwide to address specific indicators, such as the Sedentary Behavior Research Network and the Global Observatory for Physical Education [40, 41]. Additionally, some multi-country efforts have been launched for physical fitness, such as EUFITMOS and FitBack [23, 24], as well as benchmarking physical fitness against international norms in country physical activity report cards endorsed by Active Healthy Kids Global Alliance [42] or a repository compiling datasets of physical fitness testing called MO|RE [43]. Furthermore, international progress has been made in identifying research priorities and experts in physical fitness worldwide [6]. Therefore, it is timely to develop a Global Observatory of Physical Fitness to reduce the knowledge gap, harmonize fitness test batteries, and improve fitness surveillance among children and adolescents.

It is important to recognize the limitations and strengths of this scoping review, including the possibility of not capturing all available systems. Even following a systematic process of literature reviewing, our search was limited to bibliographic databases and the grey literature. In addition, although we gathered a group of experts with extensive topical knowledge, it is important to emphasize that their opinion does not represent consensus, and there may be issues that were not contemplated in the process. For instance, the expert panel identified economic and priority-related challenges and barriers to the implementation of surveillance/monitoring systems. However, since panel members were mostly from upper-middle- and high-income countries, they may not be aware of the barriers faced in lower-middle- and low-income countries. Therefore, it is important to consult and collaborate with local stakeholders and decision makers from these countries to create context-specific strategies for the implementation of their systems. Nevertheless, the comprehensive steps taken to identify and analyze the most relevant systems at the time add to the importance of this global inventory of youth fitness surveillance/monitoring systems and provide unique and innovative evidence for debate and planning. Additionally, the current review captured other fitness testing programs that did not meet the inclusion criteria but allow fitness testing mapping globally. Moreover, this review represents the first to analyze surveillance/monitoring systems for physical fitness among children and adolescents.

## Conclusion

In conclusion, we identified 15 national-level surveillance/monitoring systems for physical fitness among children and adolescents worldwide. The findings from the experts’ consultation highlight the importance of government support, adequate funding, and prioritization of fitness surveillance on the public health agenda. In addition, most surveillance/monitoring systems are linked to different governmental actions to promote fitness surveillance or physical activity practice among youth. We found a gap in monitoring among low- and lower-middle-income countries and that existing initiatives in these countries are limited to single cross-sectional studies and test batteries. Furthermore, the lack of standardization and a universal battery of tests makes it difficult to compare the results of different surveillance systems, as well as limiting the establishment of recommendations for intervention actions. By addressing the identified challenges and leveraging international collaborations, policymakers can harness the potential of fitness surveillance systems to inform evidence-based interventions and promote the physical health and well-being of young people worldwide. With the aim of reducing the knowledge gap, harmonizing fitness test batteries, and improving fitness surveillance among children and adolescents, a Global Observatory of Physical Fitness is proposed.

### Supplementary Information

Below is the link to the electronic supplementary material.

Supplementary file1 (DOCX 68 kb)

## Declarations

### Availability of Data and Materials

The data analyzed in this article are available from the corresponding author upon reasonable request.
