# Supplementary material for: Physical Fitness Surveillance and Monitoring Systems Inventory for Children and Adolescents: A Scoping Review with a Global Perspective
Source: Sports Med. 2024 May 6;54(7):1755–69. doi: 10.1007/s40279-024-02038-9 (PMC11258155; doi:10.1007/s40279-024-02038-9)
Supplement: Supplementary file 1 — Supplementary file1 (DOCX 68 kb) [file 40279_2024_2038_MOESM1_ESM.docx]

**ELECTRONIC SUPPLEMENTARY MATERIAL APPENDIX S1**

**Article title:** Physical fitness surveillance and monitoring systems inventory for children and adolescents: A scoping review with a global perspective; **Journal name:** Sports Medicine; **Author names and affiliations:** Javier Brazo-Sayavera (Universidad Pablo de Olavide), Danilo R. Silva (Universidad Pablo de Olavide), Justin J. Lang (Public Health Agency of Canada), Grant R. Tomkinson (University of South Australia), Cesar Agostinis-Sobrinho (Klaipeda University), Lars Bo Andersen (Western Norway University of Applied Sciences), Antonio García-Hermoso (Universidad Pública de Navarra), Anelise R. Gaya (Universidade Federal do Rio Grande do Sul), Gregor Jurak (University of Ljubljana), Eun-Young Lee (Queen’s University), Yang Liu (Shanghai University of Sport), David R. Lubans (University of Newcastle), Anthony Okely (University of Wollongong), Francisco B. Ortega (University of Granada), Jonatan R. Ruiz (University of Granada), Mark S. Tremblay (CHEO Research Institute), and Leandro dos Santos (Universidad Pablo de Olavide); **E-mail address of the corresponding author:** [jbsayavera@upo.es](mailto:jbsayavera@upo.es).

**Appendix S1.** Search strategy.

| **#** | **Searches** |
| --- | --- |
| 1 | child [MeSH Terms] |
| 2 | children [Title/Abstract] |
| 3 | adolescent [Title/Abstract] |
| 4 | infant [Title/Abstract] |
| 5 | 1 OR 2 OR 3 OR 4 |
| 6 | physical fitness [MeSH Terms] |
| 7 | fitness [Title/Abstract] |
| 8 | exercise [Title/Abstract] |
| 9 | strength [Title/Abstract] |
| 10 | muscle strength [Title/Abstract] |
| 11 | physical endurance [Title/Abstract] |
| 12 | cardiorespiratory fitness [MeSH Terms] |
| 13 | 6 OR 7 OR 8 OR 9 OR 10 OR 11 OR 12 |
| 14 | surveys and questionnaires [MeSH Terms] |
| 15 | surveillance [Title/Abstract] |
| 16 | surveys [Title/Abstract] |
| 17 | 14 OR 15 OR 16 |
| 18 | 5 AND 13 AND 17 |

**ELECTRONIC SUPPLEMENTARY MATERIAL APPENDIX S2**

**Article title:** Physical fitness surveillance and monitoring systems inventory for children and adolescents: A scoping review with a global perspective; **Journal name:** Sports Medicine; **Author names and affiliations:** Javier Brazo-Sayavera (Universidad Pablo de Olavide), Danilo R. Silva (Universidad Pablo de Olavide), Justin J. Lang (Public Health Agency of Canada), Grant R. Tomkinson (University of South Australia), Cesar Agostinis-Sobrinho (Klaipeda University), Lars Bo Andersen (Western Norway University of Applied Sciences), Antonio García-Hermoso (Universidad Pública de Navarra), Anelise R. Gaya (Universidade Federal do Rio Grande do Sul), Gregor Jurak (University of Ljubljana), Eun-Young Lee (Queen’s University), Yang Liu (Shanghai University of Sport), David R. Lubans (University of Newcastle), Anthony Okely (University of Wollongong), Francisco B. Ortega (University of Granada), Jonatan R. Ruiz (University of Granada), Mark S. Tremblay (CHEO Research Institute), and Leandro dos Santos (Universidad Pablo de Olavide); **E-mail address of the corresponding author:** [jbsayavera@upo.es](mailto:jbsayavera@upo.es).

**Appendix S2.** Expert panel.

| **Researcher** | **Country** | **Scholarly output** | **h-index** | **Citation count (thousands)** | **International Collaboration Impact** |
| --- | --- | --- | --- | --- | --- |
| Andersen, Lars B. | Norway | 377 | 75 | 45,2 | 149.4 |
| García-Hermoso, Antonio | Spain | 311 | 39 | 6,0 | 22.0 |
| Gaya, Reis Anelise | Brazil | 102 | 14 | 1,1 | 9.9 |
| Jurak, Gregor | Slovenia | 89 | 18 | 12,1 | 234.3 |
| Lang, Justin J. | Canada | 80 | 26 | 13,4 | 242.4 |
| Lee, Eun-Young | Canada | 77 | 18 | 3,6 | 61.8 |
| Liu, Yang | China | 47 | 17 | 1,6 | 45.6 |
| Lubans, David R. | Australia | 335 | 60 | 16,2 | 51.9 |
| Okely, Anthony D. | Australia | 382 | 64 | 17,0 | 44.2 |
| Oyeyemi, Adewale L. | Nigeria | 103 | 25 | 2,7 | 29.8 |
| Said, Mohamed A. | Saudi Arabia | 20 | 7 | 0,2 | 6.7 |
| Sardinha, Luís B. | Portugal | 414 | 68 | 30,6 | 94.7 |
| Starc, Gregor | Slovenia | 89 | 29 | 13,4 | 240.7 |
| Tomkinson, Grant R. | Australia | 133 | 31 | 4,7 | 36.6 |
| Tremblay, Mark S. | Canada | 574 | 99 | 40,6 | 84.4 |

Notes: Data retrieved from Scopus (SciVal tool) in October 2023. Scholarly output: number of published items; h-index: author-level metric that measures both the productivity and citation impact of the publications; citation count: number of cites (excluding self-cites) received to the scholarly output; international collaboration impact: metric that involves analyzing the number of international collaborations in a research output, the number of citations received by the output, and the field-weighted citation impact of the output.

**ELECTRONIC SUPPLEMENTARY MATERIAL APPENDIX S3**

**Article title:** Physical fitness surveillance and monitoring systems inventory for children and adolescents: A scoping review with a global perspective; **Journal name:** Sports Medicine; **Author names and affiliations:** Javier Brazo-Sayavera (Universidad Pablo de Olavide), Danilo R. Silva (Universidad Pablo de Olavide), Justin J. Lang (Public Health Agency of Canada), Grant R. Tomkinson (University of South Australia), Cesar Agostinis-Sobrinho (Klaipeda University), Lars Bo Andersen (Western Norway University of Applied Sciences), Antonio García-Hermoso (Universidad Pública de Navarra), Anelise R. Gaya (Universidade Federal do Rio Grande do Sul), Gregor Jurak (University of Ljubljana), Eun-Young Lee (Queen’s University), Yang Liu (Shanghai University of Sport), David R. Lubans (University of Newcastle), Anthony Okely (University of Wollongong), Francisco B. Ortega (University of Granada), Jonatan R. Ruiz (University of Granada), Mark S. Tremblay (CHEO Research Institute), and Leandro dos Santos (Universidad Pablo de Olavide); **E-mail address of the corresponding author:** [jbsayavera@upo.es](mailto:jbsayavera@upo.es).

**Appendix S3.** Complete set of questions asked of experts on surveillance/monitoring systems for physical fitness among children and adolescents.

| **#** | **Question** |
| --- | --- |
| 1 | Has any physical fitness surveillance system in children and/or adolescents not been included in Table 1? If so, tell us where to find information about it (web page, published study, others). |
| 2 | Do you have collaboration in any of the systems presented? (Yes / No) |
| 3 | Which system(s) do you collaborate on? |
| 4 | What was (is) your contribution (s) to the system? |
| 5 | From your point of view, what are (were) the main difficulties and/or barriers to the creation and implementation of a surveillance system? |
| 6 | The development of physical fitness surveillance systems was considered one of the top 10 research priorities on the health of children and adolescents for the coming years (Lang et al. 2022). What is the relevance of physical fitness surveillance systems for the health of children and adolescents and what are the main challenges for this to become a reality? |
| 7 | Please indicate below experts on the subject who can help us in the search for more surveillance systems. |

**ELECTRONIC SUPPLEMENTARY MATERIAL APPENDIX S4**

**Article title:** Physical fitness surveillance and monitoring systems inventory for children and adolescents: A scoping review with a global perspective; **Journal name:** Sports Medicine; **Author names and affiliations:** Javier Brazo-Sayavera (Universidad Pablo de Olavide), Danilo R. Silva (Universidad Pablo de Olavide), Justin J. Lang (Public Health Agency of Canada), Grant R. Tomkinson (University of South Australia), Cesar Agostinis-Sobrinho (Klaipeda University), Lars Bo Andersen (Western Norway University of Applied Sciences), Antonio García-Hermoso (Universidad Pública de Navarra), Anelise R. Gaya (Universidade Federal do Rio Grande do Sul), Gregor Jurak (University of Ljubljana), Eun-Young Lee (Queen’s University), Yang Liu (Shanghai University of Sport), David R. Lubans (University of Newcastle), Anthony Okely (University of Wollongong), Francisco B. Ortega (University of Granada), Jonatan R. Ruiz (University of Granada), Mark S. Tremblay (CHEO Research Institute), and Leandro dos Santos (Universidad Pablo de Olavide); **E-mail address of the corresponding author:** [jbsayavera@upo.es](mailto:jbsayavera@upo.es).

**Appendix S4.** Excluded surveys systems, fitness repositories, or datasets.

| **Title** | **Acronym** | **Start Year** | **Final Year** | **Country** | **Design** | **Age range** | **Measures of physical fitness** | **Frequency** | **Reasons for exclusions** |
| --- | --- | --- | --- | --- | --- | --- | --- | --- | --- |
| Danish Youth and Sport Study | DYSS | 1983 | 1991 | Denmark | Mixed longitudinal (a group of 305 were followed longitudinally, a cross-sectional sample of 1200 subjects was repeated) | 15–19 | Cross-sectional: Progressive cycle ergometer test, height, weight, BMI.  Longitudinal: Progressive cycle ergometer test, Sargent jump, 4-kg ball throw, timely weightlifting, sit-ups, timely isometric position, 4x10-m shuttle run, sit-and-reach, height, weight, BMI, skinfolds (above patellae and abdominal). | N/A | Surveillance systems at international or regional level, and/or providing local data. |
| The Australian Council for Health, Physical Education and Recreation (ACHPER) National Fitness Survey | ACHPER | 1985 | 1985 | Australia | Cross-sectional | 7–15 | 1600-m run/walk, PWC-170 cycle ergometry, isometric (handgrip, arm, leg) strength, standing long jump, sit-ups, push-ups, 50-m sprint, sit-and-reach, height, weight, circumferences (arm, waist), skinfolds (biceps, triceps, subscapular, suprailiac, and abdominal). | N/A | Cross-sectional studies, cohort, or interventional studies. |
| School Physical Fitness Award Scheme | SPFAS | 1990 | - | Hong Kong | Online platform with a battery of tests that serves as a repository and rewards students who reach established levels of physical fitness | 6–18 | 6- and 9-min run/walk, handgrip strength, sit-ups, push-ups, and upper limb muscle strength, sit-and-reach, weight, height, skinfolds (calf, triceps). | Annual | Surveillance systems at international or regional level, and/or providing local data. |
| NSW Schools Physical Activity and Nutrition Survey | SPANS | 1997 | 2015 | Australia (New South Wales) | Cross-sectional (2015) | 5–16 | 20-m shuttle run and standing long jump. | N/A | Surveillance systems at international or regional level, and/or providing local data. |
| Physical Activity among Norwegian Children study | PANCS | 2005–06 | 2011–12 | Norway | Cross-sectional (PANCS1 & PANCS 2) and cohort study (PANCS2) | 6–9; 9–15 | Progressive cycle ergometer test, standing long jump, handgrip strength, sit-ups, timely isometric position (Biering-Sorenson test), height, weight, BMI, waist circumference. | Six-yar | Cross-sectional studies, cohort, or interventional studies. |
| Identification and prevention of dietary and lifestyle-induced health effects in children and infants | IDEFICS | 2007 | 2010 | 8 countries | Cohort study | 2–9 | 20-m shuttle-run, handgrip strength, standing long jump, back saver sit-and-reach, 40-m sprint, flamingo balance. | Annual | Cohort study that included data from eight European countries. |
| Prevalence of overweight and obesity among Portuguese youth: A study in a representative sample of 10–18-year-old children and adolescents | - | 2011 | 2011 | Portugal | Cross-sectional | 10–18 | 20-m shuttle run, standing long jump, 40-m maximal velocity, height, weight. | N/A | Cross-sectional studies, cohort, or interventional studies. |
| Healthy Physical Activity Data | DAFIS | 2012 | - | Spain (Galicia) | Regional Surveillance System | 6–18 | 20-m shuttle run, handgrip strength, standing long jump, back saver sit-and-reach, 4x10-m shuttle run, bent hang arm, height, weight, waist and hip circumference. | N/A | Surveillance systems at international or regional level, and/or providing local data. |
| NHANES National Youth Fitness Survey | NNYFS | 2012 | 2012 | USA | Cross-sectional | 3–15 | Cardiorespiratory (treadmill), handgrip strength, lower body muscle strength (dynamometer), modified pull-ups, test gross motor development (TGMD-2), height, weight, upper arm length, arm circumference, waist circumference, maximal calf circumference, skinfolds (triceps, subscapular, calf). | N/A | Cross-sectional study, carried out in a single year. |
| Physical Activity and Fitness in China—The Youth Study | PAFCTYS | 2016 | N/A | China | Cross-sectional | N/A | 8x50-m shuttle run, vital capacity of lung (spirometry), standing long jump, pull-ups, timed sit-ups, 50-m sprint, timed rope-skipping, sit-and-reach, BMI. | Annual | Cross-sectional study, carried out in a single year. |
| Physical evaluation program for Uruguayan boys and girls | - | 2017 | 2017 | Uruguay | Cross-sectional | 11–14 | Standing long jump, high jump, 4x10-m shuttle run, seat-and-reach, height, weight, sitting height, wingspan, waist circumference. | N/A | Cross-sectional study, carried out in a single year. |
| European Network for the Support of Development of Systems for Monitoring Physical Fitness of Children and Adolescents | FitBack | 2022 | - | 17 countries | FitBack is a collaborative network for monitoring the physical fitness of children and adolescents | - | 20-m shuttle run, handgrip strength, standing long jump, height, weight, waist circumference. | N/A | Characterized as an international collaboration network, it is not a surveillance system. |

Abbreviations: kg = kilogram; m = meter.
